# Distribution and Localization of Mahogunin Ring Finger 1 in the Mouse Central Nervous System

**DOI:** 10.3390/ijms23168956

**Published:** 2022-08-11

**Authors:** Kazuhiko Nakadate, Kiyoharu Kawakami

**Affiliations:** Department of Basic Science, Educational and Research Center for Pharmacy, Meiji Pharmaceutical University, 2-522-1 Noshio, Kiyose 204-8588, Tokyo, Japan

**Keywords:** ubiquitin–proteasome system, attractin, immunohistochemistry, immunoelectron microscopy, MGRN1, EGF-like motif, CUB domain, C-type lectin domain, E3 ubiquitin ligase

## Abstract

Mahogunin ring finger 1 (MGRN1), an E3 ubiquitin, is involved in several physiological and neuropathological processes. Although *mgrn1* mRNA is widely distributed in the central nervous system (CNS), detailed information on its cellular and subcellular localization is lacking and its physiological role remains unclear. In this study, we aimed to determine the distribution of MGRN1 in the mouse CNS using a newly produced antibody against MGRN1. We found that the MGRN1 protein was expressed in most neuronal cell bodies. An intense MGRN1 expression was also observed in the neuropil of the gray matter in different regions of the CNS, including the main olfactory bulb, cerebral cortex, caudate, putamen, thalamic nuclei, hypothalamic nuclei, medial eminence, superior colliculus, hippocampus, dentate gyrus, and spinal cord. Contrastingly, no MGRN1 expression was observed in glial cells. Double fluorescence and immunoelectron microscopic analyses revealed the intracellular distribution of MGRN1 in pre-synapses and near the outer membrane of the mitochondria in neurons. These findings indicate that MGRN1 is more widely expressed throughout the CNS; additionally, the intracellular expression of MGRN1 suggests that it may play an important role in synaptic and mitochondrial functions.

## 1. Introduction

The ubiquitin–proteasome system (UPS) plays an important role in neuronal homeostasis [1,2,3,4,5,6,7,8]. A dysfunction in the UPS leads to the accumulation of misfolded or unassembled proteins, which contributes to neurodegeneration. Loss-of-function mutations in the genes encoding parkin, an E3 ubiquitin ligase, are linked to juvenile Parkinsonism [9,10,11,12]. The UBE3A/E6-AP protein, which is mutated in Angelman syndrome, is also an E3 ubiquitin ligase [13,14].

Mahogunin ring finger 1 (MGRN1) is an interesting new gene (RING) containing protein with E3 ubiquitin ligase activity; it is widely distributed in various tissues in mice [15,16]. MGRN1 interacts with GP78, an endoplasmic reticulum E3 ligase, and uses proteasomes to alleviate mitophagy via calmodulin as the adapter protein [17,18]. The MGRN1-mediated ubiquitination of alpha-tubulin regulates microtubule stability and mitotic spindle positioning in mitotic cells [19]. MGRN1 expression levels vary depending on the tissue; the brain, for example, has abundant MGRN1 [15,16,20]. A null mutation in MGRN1 results in a specific neuropathological change, spongiform encephalopathy [21]. Although endogenous substrate(s) for MGRN1 have not been identified, it has been suggested that MGRN1 is closely linked to the cytoplasmic tail of attractin (Atrn), a transmembrane protein containing EGF-like motifs, as well as a CUB domain, a C-type lectin domain, and a domain homologous with the ligand-binding region of the common cytokine chain [16,22,23,24]. Interestingly, rats and mice carrying loss-of-function mutations of Atrn, zitter, and mahogany also exhibit similar neuropathological phenotypes [25,26,27,28]. In situ hybridization studies have revealed similar distribution patterns of *Atrn* and *MGRN1* mRNA expression in the mouse brain [21,29]. This morphological evidence supports the hypothesis that *Atrn* and *MGRN1* are involved in the clearance of an unknown substrate whose accumulation leads to spongiform encephalopathy [16,21].

In a previous study using a specific Atrn antibody, we found that Atrn was widely distributed throughout the neuropil of the adult rodent central nervous system (CNS); we further demonstrated that *Atrn* was expressed in the neurons and in several types of glia [30]. This observation supports the view that the glia, in addition to neurons, are involved in spongiform degeneration in *Atrn*-mutant rodents. Although an in situ hybridization study delineated *MGRN1* mRNA expression in the adult mouse brain [21], this report only showed an overall sketch of MGRN1 distribution. In addition, the MGRN1 protein has only been analyzed for distribution in cultured cells and a few brain tissues [31]; there is no report on the range of expression in the brain. Furthermore, to our best knowledge, there are no reports on the intracellular distribution of MGRN1. Therefore, the detailed function of MGRN1, especially its association with organelles in the E3 ubiquitin ligase, remains speculative.

In the present study, a new affinity-purified specific antibody against MGRN1 was used to investigate in detail the regional and subcellular localization of MGRN1 in the mouse brain. The findings of this study will not only provide a basis for identifying the functional site of the molecule involved in the survival and maintenance of nerve cells, but will also help analyze the mechanism of the UPS and the role of MGRN1 in it.

## 2. Results

### 2.1. Specificity of Antibodies

**Figure 1:**
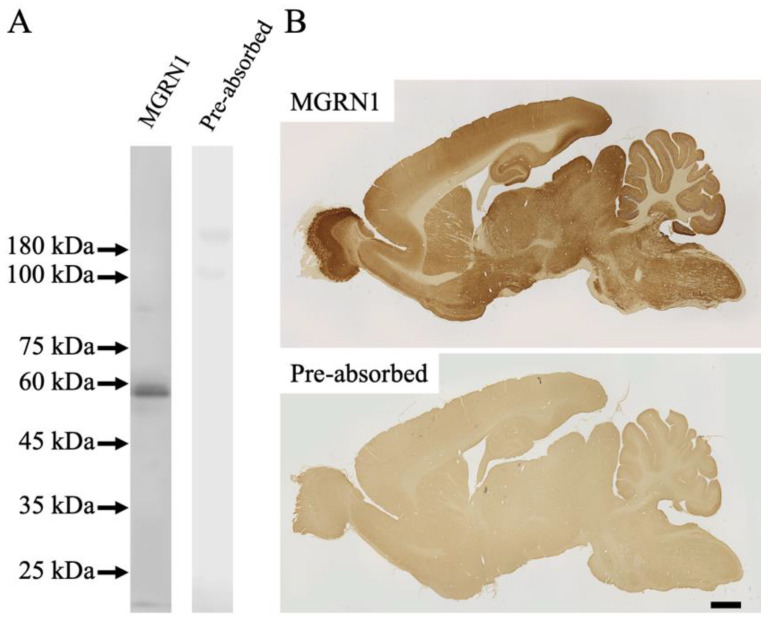
(**A**) Anti-MGRN1 antibody recognized a single band with a molecular weight of 50–60 kDa within the mouse brain membrane fraction (MGRN1 lane). No immunoreactivity was observed in Western blots generated with serum that was pre-absorbed with the synthetic peptide of the epitope (pre-absorbed lane). (**B**) Anti-MGRN1 antibody-detected immunoreactivity in the sagittal sectioned mouse brain as assessed via immunohistochemical staining (MGRN1). No immunoreactivity was observed with serum that was pre-absorbed with the synthetic peptide of the epitope (pre-absorbed). Scale bar in (**B**) = 1 mm.

The specificity of affinity-purified antibodies against MGRN1 was assessed via the immunoblot analysis of an 8-week-old mouse brain (*n* = 3) (Figure 1). The anti-MGRN1 antibody labeled a single band with a molecular weight of 50–60 kDa (the “MGRN1” lane in Figure 1A). This molecular mass weight was considered effective, based on the gene reports (NCBI reference sequence: NP_001365941.1). Immunoreactivity (IR) was completely abolished upon the pre-incubation of primary antibodies with excess amounts of the respective epitope peptides (the “pre-absorbed” lane in Figure 1A). Antibody specificity was further validated with an immunohistochemical staining experiment via the pre-incubation of the anti-MGRN1 antibody with a synthetic immunogen peptide (*n* = 3) (the “pre-absorbed” lane in Figure 1B).

### 2.2. Overview of MGRN1 Distribution

**Figure 2:**
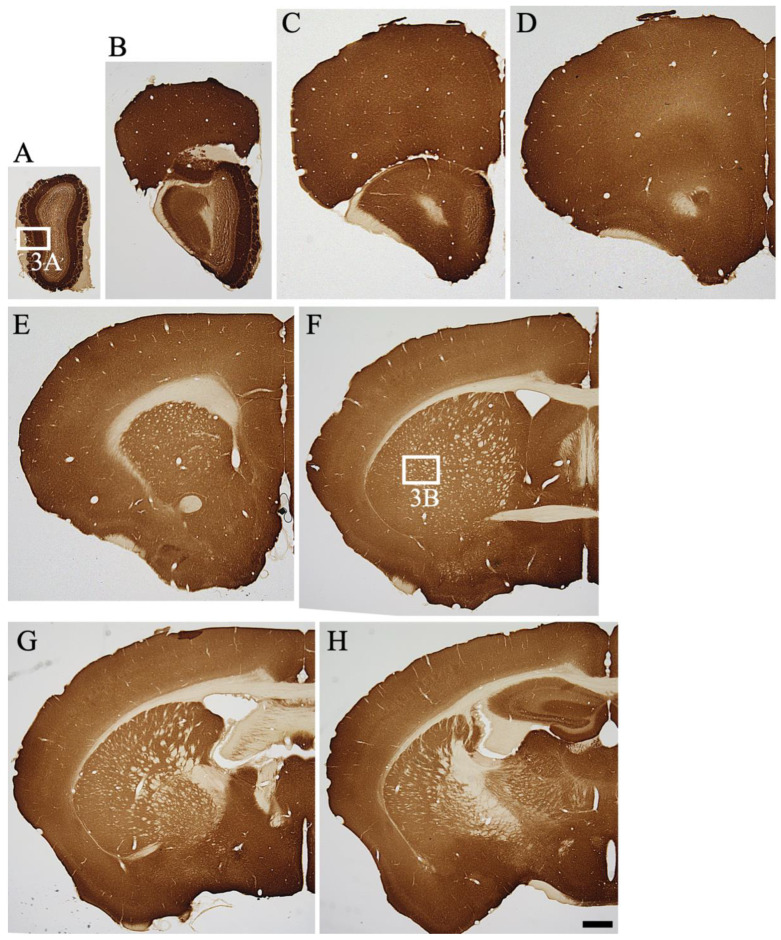

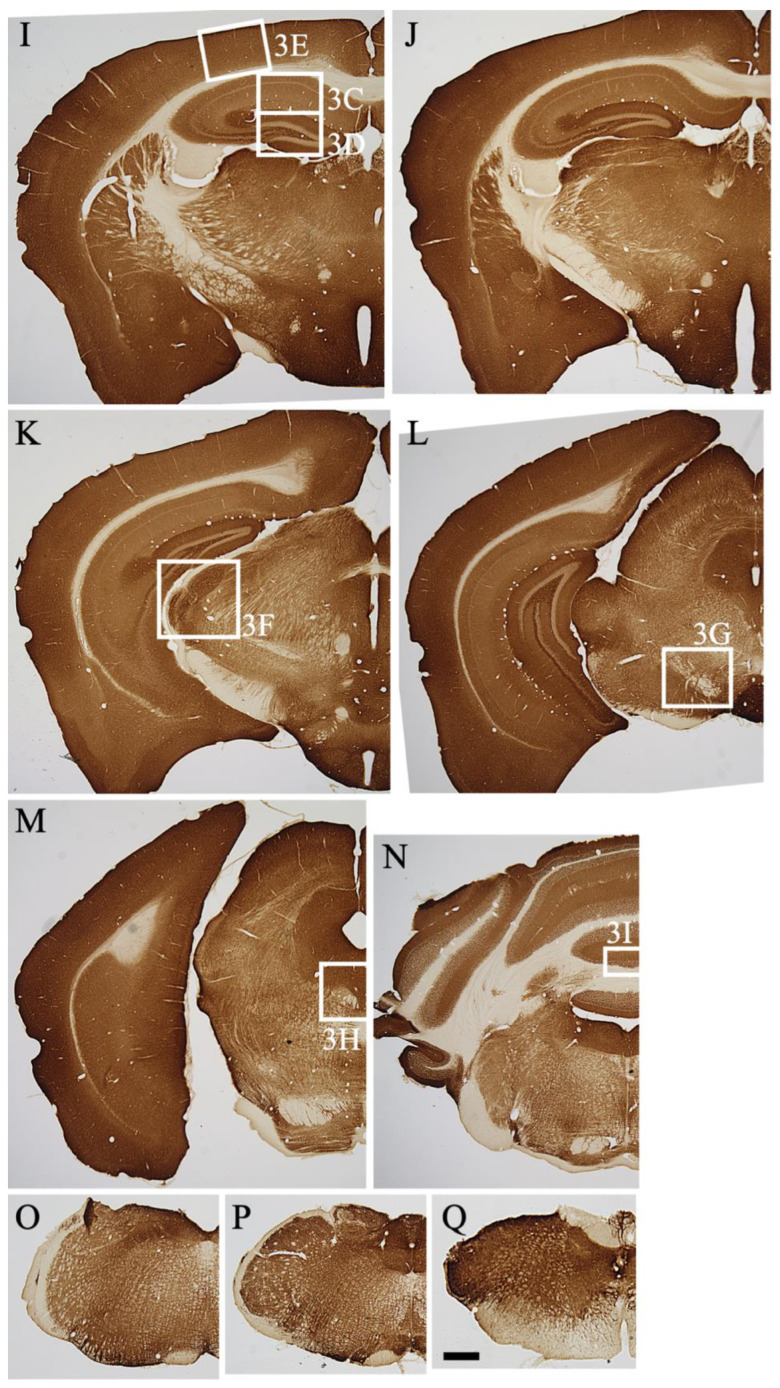
(**A**–**Q**) Immunoreactivity of MGRN1 in coronal sectioned mouse brain along the rostro-caudal axis is shown. The white boxes indicate the positions of the high magnification images shown in Figure 3. The scale bars in (**H**,**Q**) are 1 mm.

**Figure 3:**
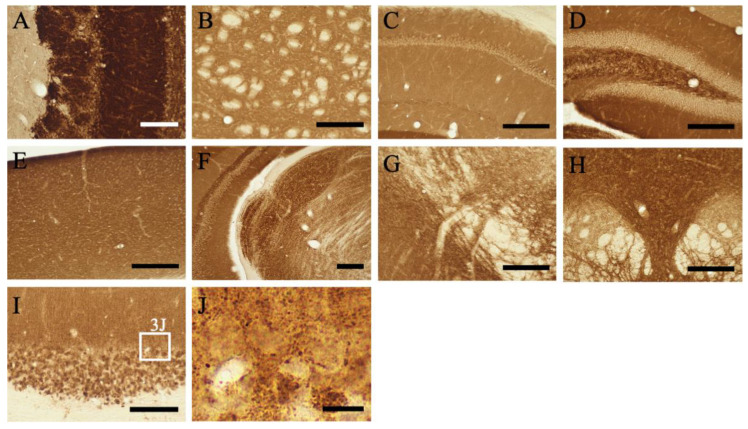
(**A**–**I**) Higher magnification views of the MGRN1 immunoreactivity shown in Figure 2 are displayed. (**J**) A higher magnification view of the white box in (**I**). The white boxes indicate the positions of the high magnification image in (**J**). The scale bars in (**A**,**I**) = 100 μm, (**B**–**H**) = 200 μm, and (**J**) = 10 μm, respectively.

The overall distribution patterns of MGRN1 were examined in the parasagittal brain sections of mice (Figure 1B). The precise anatomical localization of MGRN1 was assessed with ICR mice sections using immunohistochemical staining (*n* = 3) (Figure 2 and Figure 3). An abundant MGRN1 expression was observed in the olfactory bulb, anterior olfactory nucleus, olfactory tubercle, rostral migratory stream, cerebral cortex, hippocampus, basal ganglia, superior colliculus, and cerebellar cortex (Figure 1B). MGRN1 immunoreactivity (MGRN1-IR) was mainly located in the gray matter, which exhibited more intense MGRN1-IR than the white matter. However, MGRN1-IR in the gray matter was not distributed in the neuronal somata; most of it was present in the neuropil.

### 2.3. Detailed Distribution of MGRN1-IR in the Mouse CNS

The intensity of MGRN1-IR in the CNS region is summarized in Table 1. Among the various distinct regions, the distribution patterns of MGRN1-IR were classified into two types: the cell type, indicating MGRN1-IR located within neuronal cell bodies; and the neuropil type, indicating MGRN1-IR located in the neuropil, presumably pre-synapses.

#### 2.3.1. Telencephalon

*Olfactory bulb*: In the main olfactory bulb, intense MGRN1-IR was observed in the periglomerular, mitral, and granular cells (Figure 2A,B). The intensity of the Atrn-IR expression in the neuropil of the olfactory glomeruli varied. A moderate IR expression was observed in the neuropil of the olfactory glomeruli and external plexiform layer, but the neuropil of the granular cell layer showed low MGRN1-IR. Vigorous MGRN1-IR was observed in the vomeronasal nerve layer and the associated terminal area, the accessory olfactory bulb (Figure 2B).

*Cerebral Cortex*: MGRN1-IR showed a layer-specific pattern of expression in the cerebral cortex, irrespective of the area (Figure 2B–M). Almost all neurons in the cerebral cortex were weakly labeled with the anti-MGRN1 antibody. In particular, medium- to large-sized pyramidal neurons exhibited low MGRN1-IR. In contrast, the neuropil staining in all layers (layers I–VI) was intense.

*Hippocampus*: MGRN1-IR in the hippocampal formation also displayed a lamina-specific distribution. Pyramidal neuronal somata in CA1–3 showed low MGRN1-IR. In the stratum radiatum layers of CA1–2 and the stratum lucidum of CA3, MGRN1-IR was intense. The dentate gyrus (DG) exhibited intense staining, except in the neuronal cell layer (Figure 2H–L and Figure 3D). Abundant neuropil staining was observed in the molecular layer of the DG whereas the polymorphic layer (hilus) showed cell-specific and weak to moderate neuropil staining. In Ammon’s horn, moderate-density MGRN1-IR was observed in the neuropil of the stratum oriens and stratum radiatum.

*Fimbria and Corpus Callosum*: The MGRN1-IR staining patterns in the fimbria and corpus callosum were similar (Figure 2F–J). In these white matter structures, only a few oligodendrocytes showed MGRN1-IR; most oligodendrocytes did not express MGRN1. The subfornical organ also showed dense MGRN1-IR with a punctate appearance.

*Basal Forebrain and Septal Area*: The gray matter of the caudate putamen (CPu) showed relatively strong neuropil staining (Figure 2E–H and Figure 3B). In the globus pallidus, moderate MGRN1-IR was observed in the neuropil. The core of the nucleus accumbens showed relatively strong MGRN1-IR in the neuropil.

*Olfactory Tubercle*: In the olfactory tubercle (Tu), strong MGRN1-IR was observed (Figure 2F). This staining pattern was continued from the pyriform cortex. The island of Calleja and the nucleus of the olfactory tract also showed strong MGRN1-IR. MGRN1-IR was intense in the lateral septal nucleus (Figure 2E,F) whereas the medial septal nucleus displayed moderate-density MGRN1.

*Amygdaloid Complex*: MGRN1-IR neuropil staining was detected in all nuclei of the amygdaloid complex. The central and basolateral nuclei showed intense MGRN1-IR.

#### 2.3.2. Diencephalon

*Hypothalamus*: Almost all neurons in the hypothalamus exhibited MGRN1-IR (Figure 2G,K). The neurons in the supraoptic, suprachiasmatic, magnocellular, and parvocellular parts of the paraventricular, arcuate, posterior hypothalamic, ventromedial hypothalamic, and caudal and posterior magnocellular nuclei displayed strong MGRN1-IR. The intensity of MGRN1-IR in the neuropil of the hypothalamus decreased toward the lateral part. The neuropil of the medial pre-optic area, periventricular hypothalamus, and suprachiasmatic and arcuate nuclei showed extremely strong MGRN1-IR. In the median eminence, MGRN1-IR was observed in both the external and internal layers.

*Thalamus*: MGRN1-IR was detected in all thalamic nuclei. In particular, the medial habenular nucleus exhibited strong staining. The medial and dorsal lateral geniculate nuclei showed extremely strong neuropil staining (Figure 2K and Figure 3F). Moderate-density neuropil staining was observed in the paraventricular, medial habenular, medial geniculate, and ventral posterolateral nuclei. At a more caudal level, the olivary pretectal nucleus also showed intense staining.

#### 2.3.3. Mesencephalon

In the midbrain, strong neuropil staining was observed in the superficial layers of the superior colliculus, periaquedactal gray, and interpeduncular nucleus. The ventral tegmental area showed strong neuropil staining. In the red nucleus, large-sized neurons showed weak MGRN1-IR, but the neuropil staining was intense.

#### 2.3.4. Pons and Medulla

In general, almost all neuropils showed MGRN1-IR. The gray matter in these areas showed neuropil staining whereas the white matter showed no staining.

At the pontine level, abundant MGRN1-IR was observed in the trapezoid body. Intense staining was observed in the locus coeruleus, motor trigeminal nucleus, principal sensory trigeminal nucleus, and dorsal raphe nucleus (Figure 2M and Figure 3H). In the retrorubular area and pedunculopontine nucleus, MGRN1-IR was observed, along with weak neuronal cell somata staining. The inferior colliculus and periaquedactal gray showed moderate neuropil staining. At the pontine level of the mesensephalic trigeminal nucleus, MGRN1-IR was observed.

At the medullary level, strong neuropil staining was observed in the spinal trigeminal, inferior olivary, and gracile nuclei as well as in the raphe pallidus nucleus. The dorsal cochlear and medial vestibular nuclei, nucleus ambiguus, and dorsal motor nucleus of the vagus showed strong staining. Intense to moderate MGRN1-IR was observed in the ventral cochlear, facial, and hypoglossal nuclei as well as in the nucleus of the solitary tract. In these nuclei, strong neuropil staining was observed. Strong neuropil staining was observed in the reticular formation. Intense neuropil staining was noted in the area postrema.

#### 2.3.5. Cerebellum

The molecular layer showed moderate MGRN1-IR in the neuropil (Figure 2N and Figure 3I,J). The cell bodies of the Purkinje cells and granule cells showed very low MGRN1-IR. Interestingly, intense MGRN1-IR was observed in the flocculus. The cerebellar glomerulus in the granular layer was intensely stained. Moderate staining was also observed in the cerebellar nuclei.

#### 2.3.6. Spinal Cord

MGRN1-IR was extensively observed throughout the spinal cord. The gray matter showed intense neuropil staining and the white matter showed low staining. Intense IR was observed in layers 1 and 2 of the dorsal horn and in the ventral horn.

### 2.4. Colocalization of MGRN1

To determine the colocalization of MGRN1-IR, we confirmed the colocalization of MGRN1-IR and cellular markers in the cerebral cortex and cerebellum using the double immunohistochemical method (*n* = 3). To determine the cell types and cellular compartments displaying MGRN1-IR, double fluorescence immunostaining was performed for MGRN1 and several cell-type markers under a confocal laser scanning microscope.

**Figure 4:**
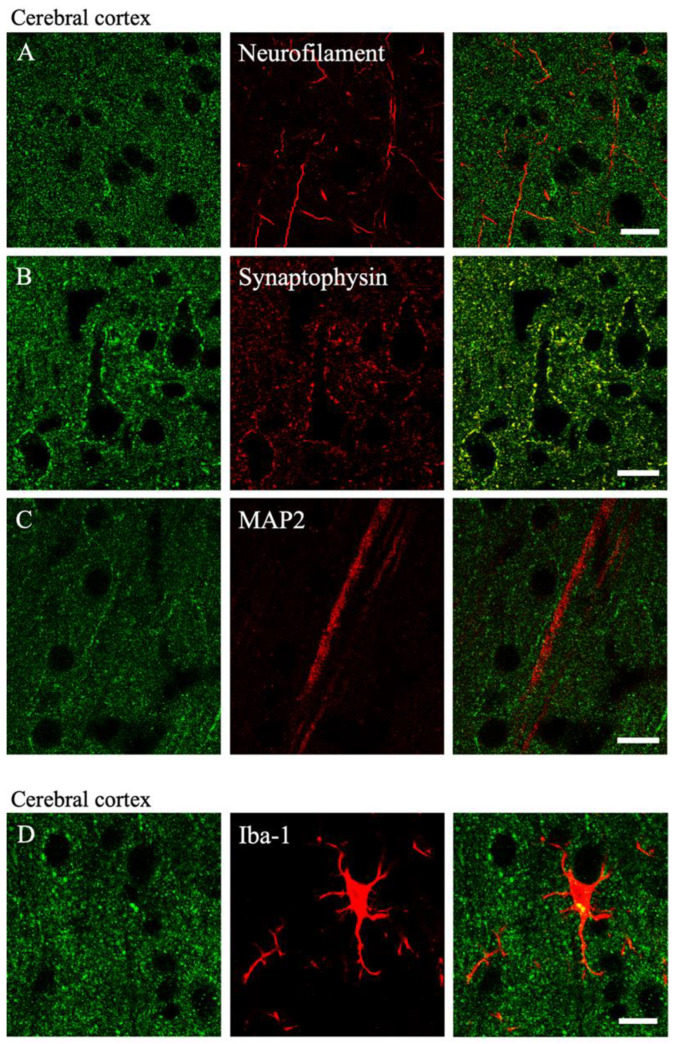

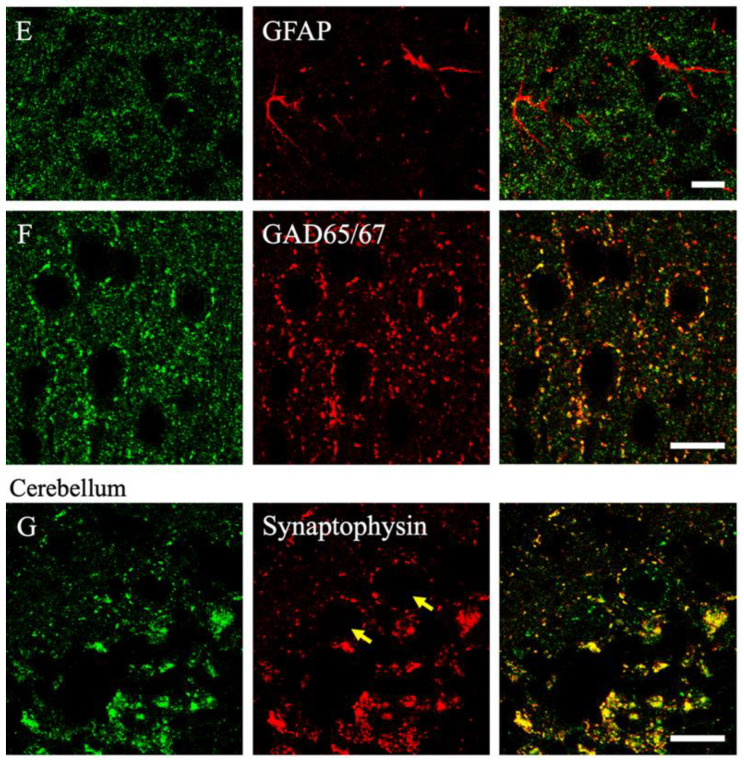
Laser scanning microscopic images of the double immunofluorescence images of the cerebral cortex (**A**–**F**) and cerebellum (**G**) are shown. Green color indicates the immunoreactivity of MGRN1 (left panels) and red color shows the immunoreactivity of each cell marker (middle panels); merged pictures are shown in the right panels. Yellow arrows in G indicate the Purkinje cells in the cerebellum. All scale bars are 10 μm.

Double staining with a neurofilament antibody barely showed any double labeling (Figure 4A). Double staining with an anti-synaptophysin antibody revealed MGRN1-IR on the pre-synaptic terminals of the cortical neurons (Figure 4B). Most terminals with MGRN1 and synaptophysin surrounded the neuronal cell somata and contacted the post-synapses. Double labeling with MGRN1 and the anti-MAP2 antibody was not observed (Figure 4C). In microglia, a low colocalization of Iba-1 and MGRN1 was found (Figure 4D). In astrocytes, a low colocalization of GFAP and MGRN1 was observed (Figure 4E). Double labeling with MGRN1 and GAD65/67 was observed in the cerebral cortex (Figure 4F). In the cerebellum, MGRN1-IR was observed in the synaptic terminals of all layers (Figure 4G).

### 2.5. Subcellular Localization of MGRN1

**Figure 5:**
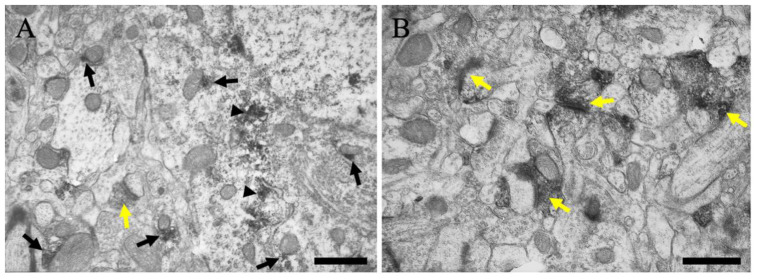
Immunoelectron microscopic views of layer V of the cerebral cortex are shown. (**A**) The neuron and neuropil near the neuronal soma are presented. (**B**) The neuropil far from the neuronal soma is presented. Black arrows indicate MGRN1 immunoreactivity near the mitochondria. Black arrowheads indicate MGRN1 immunoreactivity near the subcellular organelles without mitochondria; e.g., endoplasmic reticulum. Yellow arrows indicate MGRN1 immunoreactivity in the pre-synapses. Scale bars = 500 μm.

To determine the subcellular localization of MGRN1-IR, we analyzed the MGRN1 ultrastructural expression using immunoelectron microscopy (*n* = 3). Immunoperoxidase reaction products of MGRN1 within the neuronal perikarya in layer V of the cerebral cortex were localized near the cytoplasmic membrane of the endoplasmic reticulum (black arrowheads in Figure 5A), mitochondria (black arrows in Figure 5A), and Golgi apparatus. Furthermore, MGRN1-IR was located in the pre-synaptic terminals (yellow arrows in Figure 5A,B).

**Figure 6:**
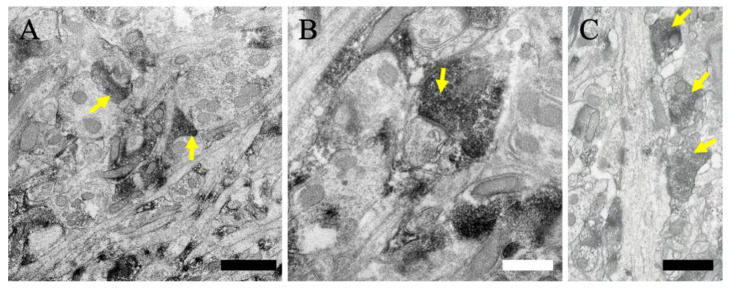

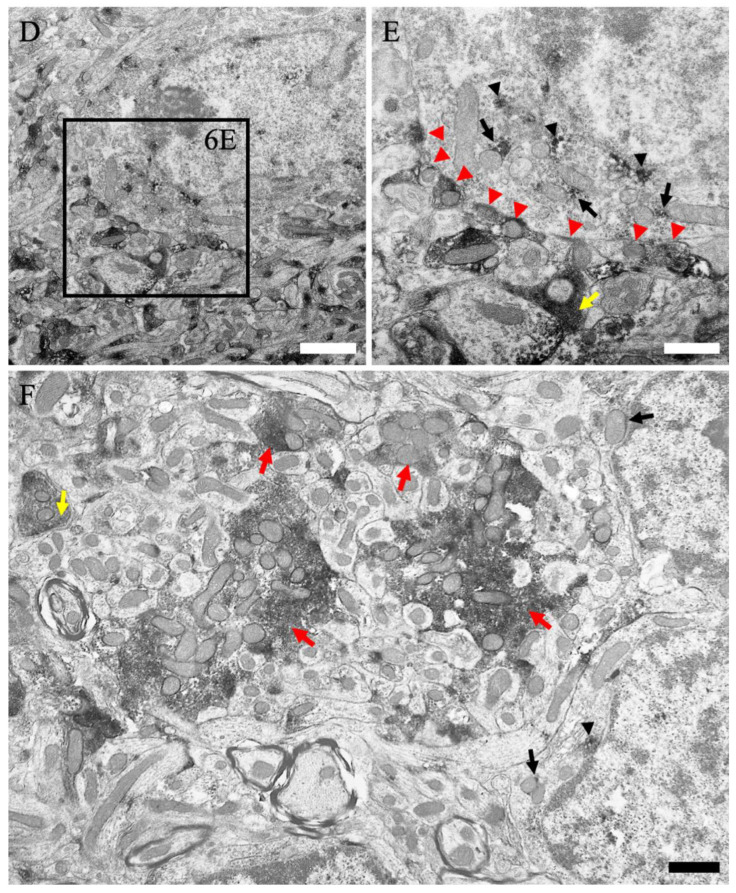
Immunoelectron microscopic views of the cerebellum are shown. (**A**–**C**) The neuropil in the molecular layer. (**D**,**E**) The Purkinje cell soma and the neuropil in the Purkinje cell layer; (**E**) shows a higher magnification view of (**D**). (**F**) The granular cell somata and neuropil in the granular cell layer. Black arrows indicate MGRN1 immunoreactivity near the mitochondria. Black arrowheads indicate MGRN1 immunoreactivity near the subcellular organelles; e.g., endoplasmic reticulum. Yellow arrows indicate MGRN1 immunoreactivity in the pre-synapses of axo-dendritic synapses. Red arrowheads indicate MGRN1 immunoreactivity in the pre-synapses of axo-somatic synapses. Red arrows indicate MGRN1 immunoreactivity in the cerebellar glomeruli. Scale bars in (**A**) and (**E**) = 1 μm; (**B**,**C**,**F**) = 500 nm; and (**D**) = 2 μm.

We then analyzed the MGRN1 localization expression in the cerebellum (*n* = 3). MGRN1-IR was located in the pre-synaptic terminals in the molecular layer (yellow arrows in Figure 6A–C). MGRN1-IR was distributed in the parallel fiber synapses (Figure 6A,B) and climbing fiber synapses (Figure 6C). In the Purkinje cell layer, immunoperoxidase reaction products in the Purkinje cell body were also localized near the cytoplasmic membrane of the mitochondria (black arrows in Figure 6E) as well as the other subcellular organelles (black arrowheads in Figure 6E). In addition, MGRN1-IR was also observed in the axo-somatic pre-synapses (black arrowheads in Figure 6E). In the granular cell layer, strong MGRN1-IR was observed in the cerebellar glomerulus (red arrowheads in Figure 6F); less MGRN1-IR was distributed in the granular cell body (black arrows and black arrowheads in Figure 6F). These labeling patterns of the MGRN1 protein observed at the electron microscopic level fully confirmed the data from the immunohistochemical and confocal laser microscopic analyses.

## 3. Discussion

### 3.1. Antibody Specificity

In this study, the distribution of MGRN1 in the mouse CNS was demonstrated. As the specificity of the anti-MGRN1 antibody in the mouse brain was unclear, we first evaluated its specificity via immunoblotting and immunohistochemical analyses. In the immunoblotting experiments, the anti-MGRN1 antibody labeled a single band with a molecular weight of 50–60 kDa; the molecular weight was as expected, based on the amino acid sequence of mouse MGRN1 (Gene Bank Accession No. 021040055.1). In the immunohistochemical analysis, a specific pattern of IR for MGRN1 was observed. As the IR was completely absorbed after the pre-incubation of primary antibodies with excess amounts of respective epitope peptides, the antibody appeared to specifically bind to the MGRN1 protein.

### 3.2. Distribution of MGRN1

The use of the newly produced antibody demonstrated that MGRN1-IR is widely distributed throughout the CNS of mice. For example, intense cell-type MGRN1-IR was observed in many distinct regions, including the olfactory bulb, caudate putamen, nucleus accumbens, central amygdaloid and basolateral amygdaloid nuclei, and medial pre-optic area as well as the paraventricular, arcuate, caudal, and posterior magnocellular, paraventricular thalamic, ventral posterolateral, dorsal lateral geniculate, and medial geniculate nuclei and also the superficial layer of the superior colliculus, interpeduncular nucleus, trapezoid body, dorsal and ventral cochlear nuclei, spinal trigeminal nucleus, raphe pallidus, nucleus ambiguus, dorsal column nuclei, and cerebellum. Widespread MGRN1-IR was observed in the neuropils of the gray matter, particularly in the sensory systems such as the olfactory system (including the olfactory bulb and piriform cortex), the visual system (including the dorsal lateral geniculate nucleus, superior colliculus, and visual cortex), the somatosensory system (including the dorsal horn of the spinal cord, spinal trigeminal nucleus, posterior column nuclei, ventroposterolateral nucleus, and somatosensory cortex), and the auditory and vestibular system (including the cochlear, vestibular, and medial geniculate nuclei). In addition to that in the sensory systems, MGRN1-IR was also observed in the motor systems such as the somatic motor system (including the somatic motor cortex and the ventral horn of the spinal cord). Although the role of MGRN1 in these sensory and motor systems still requires further investigation, physiological studies in zitter rats may help elucidate the possible roles of MGRN1 in these systems [32].

In the cerebral cortex, many neurons showed MGRN1-IR at various intensities (Figure 2 and Figure 3); however, the double immunofluorescence staining data indicated no MGRN1-IR in the neurofilament-positive axons and MAP2-positive dendrites (Figure 4). Pyramidal neurons in the cerebral cortex use excitatory amino acid transmitters and project to subcortical structures by extending axons through the white matter [33]. At the electron microscopic level, less MGRN1-IR was located in the excitatory neuronal cell bodies and many pre-synaptic terminals, but not in the axons from the white matter or post-synaptic terminals of the gray matter. Similarly, low MGRN1-IR was also observed in the GAD65/67-positive neuron cell bodies whereas strong MGRN1-IR was observed in the pre-synaptic terminals of the GABAergic neurons. Moreover, immunoelectron microscopic data from the mouse cortical neurons indicated that the intracellular domain of MGRN1 was localized in the cytoplasm near the mitochondria, Golgi apparatus, and endoplasmic reticulum (Figure 5). These results indicate that MGRN1 exists not only in the cortical excitatory neuronal network, but also in the inhibitory GABAergic neuronal network.

In the cerebellum, many neurons also showed MGRN1-IR at various intensities and MGRN1-IR was observed in the cell bodies and pre-synapses (Figure 2 and Figure 3). Double immunofluorescence staining and the immunoelectron microscopic data showed that MGRN1 was localized in the cytoplasm near the mitochondria, Golgi apparatus, and endoplasmic reticulum in both the Purkinje and granular cell bodies; additionally, intense staining for MGRN1 was observed in the pre-synapses of all layers in the cerebellum (Figure 4 and Figure 6). These findings indicate that MGRN1 plays an important role in the synaptic function in the neural circuit of the cerebellum as well as in the neural circuit in the cerebral cortex.

### 3.3. Spongiform Degeneration (Neuronal Vacuolation) and E3 Ubiquitin Ligase Activity

Spongiform degeneration is characterized by vacuolation in the nervous tissue, accompanied by neuronal death and/or gliosis. Spongiform degeneration is a common feature of neuropathological disorders such as prion diseases [34], Alzheimer’s disease [35], human immunodeficiency virus (HIV) infections [36], and Canavan’s spongiform leukodystrophy [37,38]. The same outcome of spongiform degeneration in these diseases suggests that similar cellular mechanisms must underlie the processes of spongiform change and neurodegeneration in the CNS. It has also been suggested that abnormal ubiquitination may alter the intracellular signaling and cell functions via proteasome-dependent and proteasome-independent mechanisms, leading to spongiform degeneration and neuronal cell death.

The link between aberrant ubiquitination and spongiform neurodegeneration has been strengthened by the discovery that a null mutation in the E3 ubiquitin protein ligase MGRN1 causes an autosomal recessively inherited form of spongiform neurodegeneration in animals [21]. The similarity in the spongiform degeneration phenotypes of *Mgrn1*- and *Atrn*-mutant mice suggests that MGRN1 and ATRN act in the same pathway. Recent studies have supported this hypothesis as *Mgrn1*- and *Atrn*-null-mutant mice exhibited similar defects in their mitochondrial function and increased oxidative stress to brain proteins [39,40,41]. *Atrn* overexpression reportedly protects cultured neurons against toxicity induced by the mitochondrial complex I inhibitor 1-methyl-4-phenylpyridinium (MPP+) and the proteasome inhibitor lactacystin whereas the knockdown of *Atrn* expression makes those cells more vulnerable [42]. Our previous study reported that ATRN is distributed near the mitochondrial outer membrane [30]. The present study offers the first demonstration of MGRN1 protein expression in neurons. An ultrastructural analysis revealed that the MGRN1 protein was localized near the mitochondria in the neuronal cell somata (Figure 5 and Figure 6). These distribution features indicate that the E3 ubiquitin protein ligase MGRN1 may play an important role in regulating the mitochondrial function via the ATRN signaling pathways; there are reports suggesting this possibility. In a previous study, the function of MGRN1 and the activity of cytochrome oxidase c were significantly reduced in *Atrn*-mutant mice [40].

In addition to the neurons themselves, microglia constitute a promising target. Microglia are resident immune cells in the CNS that express ATRN [30]. They are also involved in spongiform neurodegeneration [43,44]. The present study showed that they also express MGRN1. In our previous experiments, a long-lasting activation of microglia was observed throughout the brains of *Atrn*-mutant rats from 2 weeks of age [45]. Activated microglia synthesize and release several mediator substances, including a wide variety of reactive oxygen species (ROS) and inflammatory cytokines [46,47], which are toxic to neurons and glial cells [48,49,50]. Microglia, which have both MGRN1 and ATRN proteins, may play important roles in the neuronal survival pathways.

It has been demonstrated that MGRN1 multi-monoubiquitinated tumor susceptibility gene 101 (TSG101) [51,52]. It was identified as an interaction between a “PSAP” motif in MGRN1 and the ubiquitin E2 variant domain of TSG101, a component of the endosomal sorting complex required for transport I (ESCRT-I) [51]. Using cultured cells, the functional depletion of MGRN1 blocked the autophagosome–lysosome fusion and alleviated autophagic flux and its degradative competence [52]. Moreover, TSG101-null-mutant mice developed the same severe spongiform encephalopathy as *MGRN1*-mutant mice [53]. Therefore, we believe that a series of signal cascades of ATRN–MGRN1–TSG101 is involved in one of the pathogenic mechanisms of neuronal vacuolar degeneration.

In the present study, we first determined the distribution of MGRN1 in pre-synaptic terminals via an ultrastructural analysis (Figure 5 and Figure 6). The pre-synaptic terminals play a role in pre-synaptic neurotransmitter release. It has been reported that this pre-synaptic function, the neurotransmitter release, and the regulation of the surveillance and clearance of synaptic proteins are related to ubiquitination [54,55,56]. In a recent study, it was observed that the selective inactivation of two pre-synaptic active zone proteins, Piccolo and Bassoon, triggered the progressive loss of synaptic vesicle pools and the elimination of synaptic junctions [57]. These phenotypes were traced to the activation of Siah1 (an E3 ubiquitin ligase that binds to Piccolo and Bassoon), the hyper-ubiquitination of the synaptic vesicle proteins, and the degradation of the synaptic proteins via the proteasome and endolysosomal systems. Moreover, Bassoon-deficiency-triggered pre-synaptic autophagy requires an intact ubiquitin system, which primarily ubiquitinates the synaptic vesicle proteins through the activation of parkin and, to a lesser extent, Siah1 [58]. Therefore, it is thought to require polyubiquitination and degradation through proteasome-, endolysosomal-, or autophagy-related pathways in the pre-synaptic terminals. It was suggested that MGRN1 in the pre-synaptic terminals may also involve a similar mechanism of action.

A further elucidation of the pathogenic pathways involved in spongiform neurodegeneration should facilitate the development of novel rational therapies for treating prion diseases, HIV infection, and other spongiform degenerative disorders. This study clarified the previously unknown distribution of MGRN1 in the brain along with its intracellular distribution. This is considered the basis for elucidating the mechanism of cell vacuolar degeneration in the future. Furthermore, we suggest that it will contribute to the analysis of the ubiquitin proteasome system, which has a similar function, in the future.

## 4. Materials and Methods

### 4.1. Animals

Twelve male ICR strain mice (Charles River Lab., Yokohama, Japan; 8 weeks old) were used in this study. All animals were housed under temperature- and humidity-controlled conditions and a 12 h light/dark cycle with *ad libitum* access to food and water. All experiments were performed in accordance with the National Institute of Health Guide for the Care and Use of Laboratory Animals. The Laboratory Animal Ethics Committee of the Meiji Pharmaceutical University approved these experiments (No. 2706; 1 April 2020–2022); all efforts were made to minimize animal suffering and reduce the number of animals used in the study.

### 4.2. Production of the Anti-MGRN1 Antibody

A polyclonal antibody against MGRN1 was raised in guinea pigs against a synthetic peptide comprising amino acids 456–469 of the mouse MGRN1 sequence (RSPSSPIHEEDEEK; Gene Bank Accession No. 021040055.1). This sequence is very specific for the transmembrane form of MGRN1 and is conserved across species. The antigenic peptide sequence is the same in rat and human MGRN1. The antibodies were purified using the synthetic peptide coupled to sepharose 4B resin (Amersham Biosciences UK Ltd., Buckinghamshire, UK). The methods have been described elsewhere [59]. To confirm the specificity of each affinity-purified antibody, the antibodies were pre-absorbed with the synthetic peptides, which were used as the immunogen. The supernatant was tested using immunohistochemical and Western blot analyses as described below.

### 4.3. Western Blotting

Details of the Western blotting methods have been described previously [60]. The 8-week-old mice (*n* = 3) were deeply anesthetized and perfused through the left ventricle with ice-cold 0.1 M PBS (pH 7.4). The whole brain was rapidly removed and homogenized in a sodium dodecyl sulfate (SDS) sample buffer. The proteins were separated via 10% SDS-polyacrylamide gel electrophoresis and transferred onto a polyvinylidene difluoride membrane. These membranes were blocked using 10% (*w/v*) skimmed milk (Becton, Dickinson and Company, Franklin Lakes, NJ, USA) in PBS containing 0.1% Tween 20 for 1 h at room temperature. The membranes were then washed and incubated with a guinea pig anti-MGRN1 antibody (1:5000) for 2 h at room temperature. The membranes were washed and incubated with a horseradish peroxidase-conjugated anti-guinea pig antibody (1:2000, LI-COR Corporate, Lincoln, NE, USA) for 45 min at room temperature. The immunoreactive bands were detected using a Western PREMIUM chemiluminescent substrate (LI-COR Corporate) and a LI-COR C-DiGit chemiluminescence Western blot scanner (LI-COR Corporate).

### 4.4. Immunoperoxidase Staining

The detailed methods have been previously described [30,61]. For the immunohistochemical analysis, 8-week-old ICI mice (*n* = 6) were used. The animals were deeply anesthetized and perfused with physiologic saline, followed by perfusion with an ice-cold fixative containing 4% paraformaldehyde in 0.1 M PB (pH 7.4). The brain was removed and then post-fixed with the same fixative for 24 h at 4 °C. The brains were washed in 0.1 M PB and then sections were cut on a cryomicrotome (Leica Microsystems, Wetzlar, Germany) at a 30 μm thickness. The immunohistochemistry was performed using the free-floating method (*n* = 6). The sections were washed in phosphate-buffered saline containing 0.1% TritonX-100 (PBST) and then incubated for 90 min at room temperature in PBST containing 1% hydrogen peroxide. After several washings, the sections were incubated with a blocking solution containing 10% Block-Ace (Dainihon Seiyaku, Tokyo, Japan) in PBST for 2 h at room temperature and then incubated for 48 h at 4 °C with the anti-MGRN1 antibody (1:3000). After washing, the sections were incubated with a biotinylated goat anti-guinea pig antibody (1:1000, Vector Laboratories, Inc., Burlingame, CA, USA) for 2 h at room temperature, washed, and then allowed to react with an avidin–biotin peroxidase complex (ABC kit; Vector Laboratories) for 2 h at room temperature. The sections were subsequently incubated in 50 mM Tris-HCl (pH 7.3) containing 0.05% 3-3-diaminobenzidine tetrahydrochloride (DAB; Dojindo, Kumamoto, Japan) and 0.003% hydrogen peroxide. All sections were mounted on MAS-coated slides, dehydrated through graded concentrations of ethanol (Lemosol; FUJIFILM Wako Pure Chemical Corporation, Tokyo, Japan), and then mounted with Mount-Quick (Daido Sangyo, Toda, Japan). As controls, a few sections were incubated with a pre-immune serum or a pre-adsorbed primary antibody using the synthetic peptide. All sections were imaged using a CCD camera (BZ-X700, Keyence, Osaka, Japan).

### 4.5. Double Immunofluorescence Staining

The free-floating coronal sections (*n* = 3) were incubated with a blocking solution (mouse-on-mouse kit, Vector Laboratories) for 2 h at room temperature and then overnight at 4 °C with conjugated mixed primary antibodies. The anti-MGRN1 antibody (1:3000) was used with one of the following primary antibodies: neurofilament (marker for axons; 1:1000; Bio-Science, Emmenbrücke, Switzerland), synaptophysin (marker for pre-synaptic terminals; 1:1000; DAKO Cytomation, Carpinteria, CA, USA), anti-microtubule-associated protein 2 (MAP-2; marker for dendrites; 1:1000; Sigma-Aldrich, St. Louis, MO, USA), ionized calcium-binding adopter molecule 1 (Iba-1; marker for microglia; 1:2000; Wako, Osaka, Japan), glial fibrillary acidic protein (GFAP; marker for mature astrocytes; 1:2000; Sigma-Aldrich), glutamic acid decarboxylase 65/67 (GAD65/67; marker for GABAergic neurons; 1:1000; Stressgen Biotechnologies, Victoria, BC, Canada). The sections were washed in PBST and incubated with the conjugated secondary antibodies Alexa Fluor 488 goat anti-guinea pig IgG (1:2000; Molecular Probes, Eugene, OR, USA) and Alexa Fluor 568 goat anti-rabbit or mouse IgG (1:2000; Molecular Probes). After several washes, the sections were mounted on glass slides and immunofluorescence images were obtained using an FV500 confocal laser scanning microscope (Olympus, Tokyo, Japan).

### 4.6. Immunoelectron Microscopy

The details of the immunoelectron microscopic methods have been previously described [30,60,62]. For the immunoelectron microscopic analysis, 8-week-old ICI mice (*n* = 3) were used. The animals were deeply anesthetized and perfused with physiological saline, followed by perfusion with an ice-cold fixative containing 4% paraformaldehyde, 0.2% picric acid, and 0.05% glutaraldehyde in 0.1 M PB (pH 7.4). The brains were removed and then post-fixed with the same fixative for 24 h at 4 °C. The brains were washed in 0.1 M PB and then sections were cut on a VT1000S microtome (Leica Microsystems) at a 50 μm thickness. After cutting, the sections were cryoprotected in a solution containing 15% or 30% sucrose in 0.1 M PB. These sections were freeze-thawed and incubated in a blocking solution containing 10% normal goat serum in 0.1 M PBS for 2 h, followed by incubation with the anti-MGRN1 antibody (1:3000) in PBS containing 3% normal goat serum overnight at 4 °C. After washing, the sections were incubated with a biotinylated secondary antibody (1:100, Vector Laboratories) for the immunoperoxidase reaction and then allowed to react with ABC and DAB. After a treatment with OsO4, the sections were stained with uranyl acetate and dehydrated. The sections were then embedded in Epon-812 resin (TAAB, Berkshire, UK). Ultrathin sections were prepared using a Leica EM UC6 Ultramicrotome (Leica Microsystems) and examined using a JEM-1011 electron microscope (JEOL, Tokyo, Japan).

### 4.7. Image Processing

A densitometry analysis of IR using the ABC–DAB reaction was performed on a computer using ImageJ free software (US National Institute of Health, Bethesda, MD, USA) and the density was measured on a 5-point scale. The details of these methods have been described previously [30]. The threshold of MGRN1 intensity was set at twice the baseline value using the ImageJ software program. The following 5-point density scale was used: ++++, highest density; +++, higher density; ++, high density; +, low density; -, background density. Due to the methodological limitations, the density scale indicated the average density of the cell and neuropil staining. The anatomical structures were identified by a direct observation of the Nissl-stained sections using the atlas of Paxinos and Franklin for mice as a basic reference [63].

## Figures and Tables

**Table 1:** Immunohistochemical localization of MGRN1-IR in mouse CNS.

Brain Region				Expression	Brain Region			Expression
Olfactory						Ventral posteromedial nucleus		++
	Main olfactory bulb					Dorsal lateral geniculate nucleus		++
		Glomerular layer		++++		Intergeniculate leaflet		++
		External plxiform layer		+++		Medial geniculate nucleus		+++
		Mitral layer		-	Mesencephalon			
		Internal granular layer		++		Superior colliculus		
	Accessory olfactory bulb			++			Superficial layer	+++
	Vomeronasal nerve layer			+			Deep layer	++
Cerebral cortex						Periaquedactal gray		+++
		Layer I–VI		++ ~ +++		Oculomotor nucleus		++
Hippocampal formation						Red nucleus		+++
	Ammon’s horn (CA1–CA3)					Substantia nigra		+++
		Stratum oriens		++		Ventral tegmental area		++
		Pyramidal layer		++		Interpeduncular nucleus		+++
		Stratum radiatum		+	Pons			
		Stratum lacnosum-moleculare		+		Dosal tegmental nucleus		+++
	Dentate gyrus					Dosal raphe nucleus		+++
		Molecular layer	Outside half	+++		Median raphe nucleus		++
			Intside half	+++		Prabrachial nucleus		++
		Granular layer		++		Inferior colliculus		++
		Hillus		++++		Retrorubular area		++
Basal forbrain and septal area						Pedunculo pontine nucleus		++
	Bed nuclei of stria terminalis			++		Trapezoid body		+++
	Claustrum			++		Locus coeruleus		++
	Basal ganglia					Motor trigeminal nucleus		++
		Caudate putamen		+ ~ ++		Principal sensory trigeminal nucleus		++
		Globus pallidus		+	Medulla oblongata			
	Nucleus accumbens					Dorsal cochlear nucleus		+++
		Core		++		Ventral cochlear nucleus		+++
		Shell		+ ~ ++		Medial vestibular nucleus		+++
	Olfactory tubercle			+ ~ +++		other vestibular nucleus		++
	Island of Calleja			+++		Spinal trigeminal nucleus		+++
	Lateral septal nucleus			++		Facial nucleus		+++
	Medial septal nucleus			++		Raphe pallidus		+++
	Nucleus of lateral olfactry tract			+++		Ambiguus nucleus		+++
	Piriform cortex			++++		Nucleus of solitary tract		++
Amygdaloid complex						Dorsal motor nucleus of vagus		+++
	Central amygdaloid nucleus			++		hypoglossal nucleus		+++
	Basolateral amygdaloid nucleus			++		Inferior olive		++
	Medial amygdaloid nucleus			+++		Externsl cuneate nucleus		+++
Hypothalamus						Cuneate nucleus		+++
	Medial preoptic area			+++		Gracile nucleus		+++
	Supraoptic nucleus			+++	Cerebellum			
	Suprachiasmatic nucleus			+++		Molecular layer		+++
	Paraventricular nucleus			+++		Purkinje cell layer		++
	Periventricular nucleus			+++		Granular cell layer		+++
	Lateral hypothalamic area			+		Cerebellar nuclei		++
	Dorsal hypothalamic area			++	Spinal cord			
	Arcuate nucleus			+++		Dorsal horn		+++
	Dorsomedial hyphothalamic nucleus			++		Ventral horn		+++
	Ventromedical hypothalamic nucleus			+++	Circum ventricular organ and related area			
	Zona incerta			+			Subfornical organ	+++
	Medial mammillary nucleus			++			Median eminence	++++
	Caudal and posterior magnocellular nuclei			++				
Epithalamus and thalamus							Subcommissural organ	+++
	Medial habenular nucleus			+++			Area postrema	++++
	Lateral habenular nucleus			++			Rostral migratory stream	++
	Paraventricular thalamic nucleus			+++			Ependyma	++
	Ventral posterolateral nucleus			++				

Intensity of MGRN1-IR was evaluated by computer associated densitometry: ++++, highest density; +++, higer density; ++, high density; +, low density; -, background density.

## Data Availability

Not applicable.
